# Head-to-Tail and Head-to-Head Molecular Chains of Poly(*p*-Anisidine): Combined Experimental and Theoretical Evaluation

**DOI:** 10.3390/molecules27196326

**Published:** 2022-09-26

**Authors:** Lilian Rodrigues de Oliveira, Douglas de Souza Gonçalves, Adriano de Souza Carolino, William Marcondes Facchinatto, Diogo de Carvalho Menezes, Cleverton Oliveira Dias, Luiz Alberto Colnago, Yurimiler Leyet Ruiz, Ştefan Ţălu, Henrique Duarte da Fonseca Filho, Puspitapallab Chaudhuri, Pedro Henrique Campelo, Yvonne Primerano Mascarenhas, Edgar Aparecido Sanches

**Affiliations:** 1 Graduate Program in Physics (PPGFIS), Federal University of Amazonas, Manaus 69077-000, AM, Brazil; 2 Laboratory of Nanostructured Polymers (NANOPOL), Federal University of Amazonas, Manaus 69077-000, AM, Brazil; 3 Brazilian Corporation for Agricultural Research, EMBRAPA Instrumentation, São Carlos 13560-970, SP, Brazil; 4 Graduate Program in Materials Science and Engineering (PPGCEM—EESC), University of São Paulo (USP), São Carlos 13563-120, SP, Brazil; 5 São Carlos Institute of Chemistry (IQSC), University of São Paulo, São Carlos 13566-590, SP, Brazil; 6 The Directorate of Research, Development and Innovation Management (DMCDI), Technical University of Cluj-Napoca, 15 Constantin Daicoviciu St., 400020 Cluj-Napoca, Romania; 7 Department of Physics, Federal University of Amazonas (UFAM), Manaus 69077-000, AM, Brazil; 8 Laboratory of Synthesis of Nanomaterials and Nanoscopy (LSNN), Federal University of Amazonas, Manaus 69077-000, AM, Brazil; 9 Department of Food Technology, Federal University of Viçosa (UFV), Viçosa 36570-900, MG, Brazil; 10 São Carlos Institute of Physics (IFSC), University of São Paulo (USP), São Carlos 13563-120, SP, Brazil

**Keywords:** poly(*p*-anisidine), conjugated polymer, DFT, head-to-tail, head-to-head, multifractal analysis

## Abstract

Poly(*p*-anisidine) (PPA) is a polyaniline derivative presenting a methoxy (–OCH_3_) group at the *para* position of the phenyl ring. Considering the important role of conjugated polymers in novel technological applications, a systematic, combined experimental and theoretical investigation was performed to obtain more insight into the crystallization process of PPA. Conventional oxidative polymerization of *p*-anisidine monomer was based on a central composite rotational design (CCRD). The effects of the concentration of the monomer, ammonium persulfate (APS), and HCl on the percentage of crystallinity were considered. Several experimental techniques such as X-ray Diffraction (XRD), Scanning Electron Microscopy (SEM), multifractal analysis, Nuclear Magnetic Resonance (^13^C NMR), Fourier-transform Infrared spectroscopy (FTIR), and complex impedance spectroscopy analysis, in addition to Density Functional Theory (DFT), were employed to perform a systematic investigation of PPA. The experimental treatments resulted in different crystal structures with a percentage of crystallinity ranging from (29.2 ± 0.6)% (PPA1_HT_) to (55.1 ± 0.2)% (PPA16_HT-HH_). A broad halo in the PPA16_HT-HH_ pattern from 2θ = 10.0–30.0° suggested a reduced crystallinity. Needle and globular-particle morphologies were observed in both samples; the needle morphology might have been related to the crystalline contribution. A multifractal analysis showed that the PPA surface became more complex when the crystallinity was reduced. The proposed molecular structures of PPA were supported by the high-resolution ^13^C NMR results, allowing us to access the percentage of head-to-tail (HT) and head-to-head (HH) molecular structures. When comparing the calculated and experimental FTIR spectra, the most pronounced changes were observed in ν(C–H), ν(N–H), ν(C–O), and ν(C–N–C) due to the influence of counterions on the polymer backbone as well as the different mechanisms of polymerization. Finally, a significant difference in the electrical conductivity was observed in the range of 1.00 × 10^−9^ S.cm^−1^ and 3.90 × 10^−14^ S.cm^−1^, respectively, for PPA1_HT_ and PPA16_HT-HH_.

## 1. Introduction

The polyconjugated structures of intrinsically conducting polymers (ICPs) play an important role, mainly in their crystal structures and electrical properties [1,2,3]. Some advantages of conjugated polymers are based on their methods of synthesis [4] and good environmental stability, allowing for the development of novel materials [5,6,7].

Structural aspects in polyanilines continue to be an interesting research topic [8,9,10]. Efforts have been devoted to improving their crystallinity, processability, and electrical conductivity by using appropriate functionalized protonic acids, novel mechanisms of polymerization, and substituted polyanilines [2,4,11]. Polyaniline derivatives are based on a suitable substituent attached either at the nitrogen atom or in the phenyl ring of the repeated unit [2,12,13,14].

Anisidine is an aromatic amine (methoxyaniline) present in three isomeric forms: *ortho*-, *meta*-, and *para*-anisidine. The electronic characteristics of aniline derivatives are based on the aniline ring substitutions [15]. Among the substituted polyaniline derivatives, poly(*p*-anisidine) (PPA) is a polyaniline presenting a methoxy (–OCH_3_) group at the *para* position of the phenyl ring [16,17,18]. The electron-donating substituent groups in the aromatic rings strongly introduce the conformational modifications of conjugated polymers [2] influencing a range of physicochemical properties. Structural, morphological, thermal, and electrical properties of *ortho*- and *meta*-substituted polyanilines have been widely reported [2,4,11]. However, systematic reports on poly(*p*-anisidine) (PPA) were not found in the scientific literature. There is a significant lack of information on pure PPA [19]. However, some reports were devoted to developing PPA-based nanocomposites [17,18,20,21].

The preparation of electroactive nanocomposites based on PPA and clay were performed using oxidative polymerization [21], and the intercalation of PPA was confirmed by the increased interlayer spacing and exfoliation forms. The synthesis of PPA/TiC and PPA-co-ANI/TiC nanocomposites was also reported [18]. The authors explored the use of PPA as an alternative to polyaniline in anodic materials for hydrometallurgy. Nanocomposites were also successfully synthesized using the oxidative polymerization of *p*-anisidine and/or aniline monomers on TiO_2_ nanoparticles [20], producing electroactive microspheres. The authors proposed their use as fillers for antistatic and anticorrosion coating. Nanocomposites based on PPA and ZnO were synthesized by adding the semiconductor metal oxide to the polymeric solution [17]. An analysis of the electrochemical conductivity suggested blends with enhanced conductivity nature.

In order to evaluate the structural, morphological, spectroscopic, and electrical properties of PPA, a conventional oxidative polymerization of *p*-anisidine was carried out in the present work. The effect of the concentration of *p*-anisidine monomer, ammonium persulfate (APS), and HCl on the percentages of crystallinity of each PPA sample was considered. The X-ray Diffraction technique (XRD) was applied to examine the long-range order achieved as a consequence of very short-range interactions and to estimate the percentage of crystallinity. The ^13^C NMR experiments were useful in confirming the head-to-tail (HT) and head-to-head (HH) mechanisms of polymerization of PPA. Scanning Electron Microscopy (SEM) was useful in correlating the influence of the mechanisms of polymerization on the polymer morphology: a detailed and local description of complex scaling behaviors from the SEM images were obtained using multifractal analysis. Fourier-transform Infrared spectroscopy (FTIR) was applied for molecular structural characterization, as well as to confirm the mechanisms of polymerization of PPA. Density Functional Theory (DFT)-based computational approaches were employed to investigate the molecular geometry of PPA through the results obtained experimentally by using ^13^C NMR. These results were correlated with those obtained experimentally. Finally, the electrical conductivity of the developed polymers was accessed by using a complex impedance spectroscopy analysis.

## 2. Materials and Methods

### 2.1. Polymer Synthesis

Conventional oxidative polymerization of *p*-anisidine was carried out at 25 °C based on a central composite rotational design (CCRD) [22]. The number of treatments was composed of factorial, axial and central points (2^k^ + 2xk + central points), where *k* represents the number of parameters ((i) (*p*-anisidine) (g), (ii) ammonium persulfate (APS) (g), and (iii) HCl (*M*)). Table 1 shows the parameters of the syntheses.

Solution I was prepared by solubilizing the *p*-anisidine monomer (2 g) in HCl 1*M* (150 mL). Solution II, on the other hand, was obtained by adding APS (4 g) in HCl 1*M* (200 mL). Solution II was added drop-by-drop to solution I. The resulting solution was maintained under constant stirring for 3 h. Then, the dark powder was vacuum-filtered, washed with distilled water, and maintained in a desiccator until reaching a constant weight to obtain the polymer labeled as PPA1. The same methodology was performed using the parameters in Table 1 to obtain the PPAs labeled as PPA2 to PPA17.

### 2.2. XRD Analysis and Percentage of Crystallinity

XRD data were obtained on a Panalytical Empyrean diffractometer (Malvern, UK) operating with CuK_α_ radiation at 40 kV and 40 mA. Data collection was performed in the angular range of 2θ = 3–100° with a step size of 0.01° and 5 s/step. The simple area separation method [10,23] was applied to estimate the percentage of crystallinity. This method required the separation/quantification of the integrated intensities from the crystalline and noncrystalline phases. A noncrystalline XRD pattern of PPA was obtained after the heat treatment of PPA1 at 300 °C for 30 min. Then, the ratio between the peak areas to the noncrystalline broad halo was obtained using a routine software. The integration process was performed using the full XRD pattern of PPA1 to PPA17.

### 2.3. SEM Analysis

Powdered polymers were placed on a carbon tape. SEM images were taken at 25 °C on a Carl Zeiss Supra 35 microscope (Jena, Germany) using 4.0 kV.

#### Multifractal Analysis

The fractal dimension is used to specify the complexity of a fractal object by measuring the topographical variations in relation to the scale factor. The number of square cells, *N*(*ε*), in relation to the scale factor, *ε*, is expressed as:

(1) $$N\left(\varepsilon \right)\;\propto \varepsilon^{-D}$$

where *D* is the fractal dimension. So, in order to describe the size distribution of various objects, a scale (or power) law was used, which used the box counting method widely employed to determine the fractal dimension of an irregular object.

(2) $$D=\underset{\varepsilon \to 0}{\lim}\frac{\log N\left(\varepsilon \right)}{\log\varepsilon }$$

The objective of this method is to cover a fractal set with boxes of different sizes and to interpret how the number of boxes changes with respect to size [24,25]. However, fractal models are not able to clearly characterize the spatial anisotropy. On the other hand, a multifractal analysis can provide more information than a monofractal one. In this way, a detailed local description of the complex scaling behaviors in SEM images was obtained using a multifractal analysis based on a spectrum of singularity exponents. Data were extracted using computational routines [26,27,28] with the software MATLAB version 8.2.0.29 (R2013b). The multifractal system was composed of interconnected subsets with different fractal dimensions. Due to its simplicity and wide use, the method of moments was applied. Through the probability density in the *i*-th square, the mass deposition at the local level can be estimated using the following equation [29]:

(3) $$P_{i}\left(\varepsilon \right)=\frac{N_{i}\left(\varepsilon \right)}{\sum N_{i}\left(\varepsilon \right)}$$

where *N_i_*(*ε*) is the number of pixels containing mass in the *i*-th box of size ε and the denominator is the total mass of the system. This system can be characterized when its surface contains *N*(*ε*) square cells, whose statistical sum is [28,30]:

(4) $$Z\left(q,\varepsilon \right)=\overset{N\left(\varepsilon \right)}{\underset{i=1}{\sum }}P_{i}^{q}\left(\varepsilon \right)∼\varepsilon^{\tau \left(q\right)}$$

where *q* represents the order moment with real values from −∞ (less dense areas) to +∞ (dense areas). The generalized fractal dimensions (*Dq*), which correspond to scaling exponents for the *q*-th order of the measure, can be defined by (for q≠1) [31]:

(5) $$D_{q}=\frac{1}{\left(q-1\right)}\underset{\varepsilon \to 0}{\lim}\frac{\log Z\left(q,\varepsilon \right)}{\log\varepsilon }$$

When q=1, to calculate the generalized fractal dimension *D*_1_, we use:

(6) $$D_{1}=\underset{\varepsilon \to 0}{\lim}\frac{\;\sum_{i=1}^{N\left(\varepsilon \right)}P_{i}\left(\varepsilon \right)P_{i}^{q}\left(\varepsilon \right)}{\log\varepsilon }$$

The mass exponent can be obtained by the following equation:

(7) $$\tau \left(q\right)=\underset{\varepsilon \to 0}{\lim}\frac{\log Z\left(q,\varepsilon \right)}{\log\varepsilon }$$

By combining (5) and (7):

(8) $$D_{q}=\frac{\tau \left(q\right)}{\left(q-1\right)}$$

Therefore, the multifractal spectrum function can be calculated as [31]:

(9) f(α(q))=qα(q)−τ(q)

where *α*(*q*) *= dτ*(*q*)/*dq*. *Dq* and *α*(*q*) are calculated as the generalized dimension of the Hölder exponents of *q* and provide information about fractal/multifractal geometry, while *f*(*α*) is related to the singularity spectrum [32,33].

### 2.4. ^13^C NMR Spectroscopy

The high-resolution solid-state ^13^C NMR experiments were performed on a Bruker^®^ Advance 400 spectrometer using a Bruker 4–mm magic-angle spinning (MAS) double resonance probe head (Bruker, Billerica, MA, USA) operating at 400.0 MHz (^1^H) and 100.5 MHz (^13^C) with 2.5 μs and 4.0 μs of π/2 pulse length, respectively. About 200 mg of powdered samples was packaged into 3.2 mm zirconia rotors; all spectra were recorded at (25 ± 1) °C. RF–ramped cross-polarization (^13^C CPMAS) [34] and Spinal-16 high-power ^1^H decoupling [35] performed with γB1/2π = 70 kHz nutation frequency were applied for ^13^C signal acquisition. The acquisition parameters were set at 5 s of recycle delay, 40 ms of acquisition time, and 1024 scans.

### 2.5. Computational Method

Since the ^13^C NMR spectroscopy results indicated the existence of head-to-tail (HT) and head-to-head (HH) polymerization for PPA, geometry optimizations were performed for both forms of the PPA polymer structures (henceforth labeled as PPA_HT_ and PPA_HH_ for HT and HH polymerization, respectively) using quantum-chemical density functional theory [36] as implemented in the Gaussian 03 program package (Wallingford, CT, USA) [37]. In particular, the gradient-corrected correlation functional of Perdew, Burke, and Ernzerhof (PBE) [38,39] in combination with the 6-311G(d,p) basis set was utilized for this purpose. However, previous studies also reported the use of hybrid functionals in the evaluation of conducting polymers [40,41]. Both PPA_HT_ and PPA_HH_ are tetramers formed out of covalent bonding of the four *p*-anisidine monomers in the conformation of the polymer chain. The doped forms of PPA were also considered (labeled as Cl–PPA_HT_ and Cl–PPA_HH_) while taking into account the chlorine counterions in a half-oxidized PPA tetramer. Harmonic vibrational frequencies were determined for each optimized geometry at the same level of theory; positive frequencies were found for all vibrational modes in each optimized PPA to guarantee the obtention of a local minimum on the potential energy hypersurface. Preparation of the initial molecular structures and partial analysis of the calculated results were conducted with the aid of the Gaussview program [42].

### 2.6. FTIR Analysis

FTIR spectra were recorded using a Shimadzu IR Prestige-21 spectrometer (Kyoto, Japan) from 4000 to 400 cm^−1^ at a resolution of 1 cm^−1^ and using 64 scans.

### 2.7. Electrical Conductivity

A Solartron 1260 impedance analyzer was used for collecting data at 27 °C by applying 500 mV from 10 Hz to 1 MHz. Polymers were pellets (1.3 cm in diameter and 0.14 cm in thickness) without thermal treatment and conductive ink exposure.

## 3. Results and Discussion

### 3.1. Percentage of Crystallinity and XRD Analysis

The PPAs were synthesized according to the experimental conditions described in Table 1. A total of 17 syntheses were performed (PPA1 to PPA17) based on the CCRD method. However, the polymerization of PPA11 was not observed.

The crystalline state is based on a 3D positional and orientational order. Continued growth of the crystalline polymer phase results in large-scale polymerization with polymer crystals lying in certain preferred directions [43]. In polymer crystals, the macromolecules are longer than the unit cell parameters and each polymer chain is supposed to pass through several unit cells. For this reason, general requirements for the nucleation and growth of the crystalline phase of polymers are based on the regularity in the chemical constitution as well as in the configuration of long sequences of monomeric units [44]. The conformation of the polymer chains in the crystalline state depends on the configuration of the stereoisomeric centers present along the chains and is based on the principles of equivalence and of minimum internal conformational energy [45].

The three-dimensional long-range order is never present in polymer crystals; the structural disorder is a rule rather than an exception [44]. For this reason, the crystallinity concept in polymeric materials is significantly complex and is considered to be a semicrystalline material generally composed of crystals (lamellae) embedded into a noncrystalline phase, resulting in a highly interconnected network [45]. The verification of the existence of crystalline regions in polymeric materials became more evident in the 1920s, when some polymers subjected to XRD analysis presented characteristic peaks (previously observed only in crystalline materials). These peaks appeared in addition to a diffuse halo, allowing the confirmation of the coexistence between the crystalline and noncrystalline phases in polymeric materials.

The percentage of crystallinity of polymeric materials is then related to the amount of the crystalline contribution in the entire material. An absolute value is not possible to be obtained because it depends on the technique used to estimate crystallinity [46]. Furthermore, several parameters and synthesis methodology influence the polymer crystallinity, so a range of crystallinity values can be obtained for a specific polymer [4].

Herein, the calculation of the percentage of crystallinity of each *as*-synthesized PPA was based on the separation and quantification of the integrated intensities from the semicrystalline (PPA1 and PPA16) and noncrystalline (heat-treated PPA1) phases. This method was based on the obtainment of an internal diffraction pattern of the same sample in the noncrystalline state [10,47]. A completely noncrystalline polymer of the same chemical composition can be used as a standard for estimating the percentage of crystallinity [48]. This noncrystalline pattern can often be obtained by heat or chemical treatments or other methodologies, resulting in a noncrystalline material. Considering conjugated polymers, acid–base neutralization reactions were previously reported [10]. The separation and quantification of the integrated intensities from a semicrystalline state to calculate the percentage of crystallinity of polymer materials were previously reported elsewhere [10,49].

The effect of the concentration of the monomer, APS, and HCl on the percentages of crystallinity of each PPA sample was considered. The treatments performed experimentally resulted in different crystal structures due to the combination of different amounts of reagents applied in each chemical oxidative polymerization, giving rise to 16 different XRD patterns (data not shown). The percentage of crystallinity ranged from (29.2 ± 0.6)% to (55.1 ± 0.2)%.

**Figure 1:**
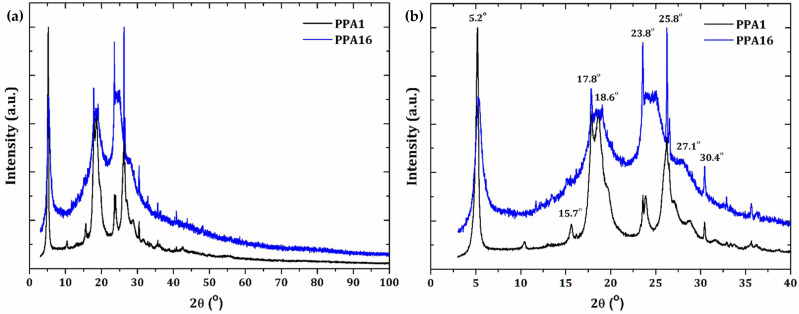
Semicrystalline XRD patterns of the (**a**) *as*-synthesized PPA1 and PPA16 and (**b**) the angular region 2θ = 3–40° highlighting the most intense diffraction peak positions.

Figure 1 shows the semicrystalline XRD patterns of the *as*-synthesized PPA1 and PPA16 as a consequence of some polymer chain alignment. However, broad peaks resulted due to the nanosized crystalline phase (crystallites) that coexisted in a noncrystalline phase.

The scientific literature has reported that several factors influenced the crystallinity and polymerization of polyaniline and its derivatives. These factors may be related to the different synthesis methodologies [50,51,52]; the regular packing of the polymer chains; the ring side group at the *ortho*, *meta,* or *para* positions [53,54,55,56]; the nature of the doping acid and counterion size [19,57]; and possible chemical or physical interactions between counterions and the ring side group [2]. Our results showed that the doping acid concentration presented the greatest effect on the percentage of crystallinity of PPA, followed by the *p*-anisidine monomer and APS. Then, only the polymers showing the highest (PPA1) and lowest (PPA16) percentage of crystallinity were selected for further analysis.

The XRD patterns of PPA1 and PPA16 were clearly correlated. Both patterns presented an intense peak at 2θ = 5.2° (*d* = 16.9 Å). However, this peak in PPA1 was significantly narrow, probably due to the presence of larger crystallites. On the other hand, a broad halo in the PPA16 pattern from 2θ = 10.0–40.0° suggested reduced crystallinity.

The XRD pattern of PPA16 also showed two broad and intense peaks: the first one was found from 2θ = 16.5° to 21.0°, centered at 2θ = 18.5° (*d* = 4.8 Å); the second broad peak ranged from 2θ = 22.5° to 26.7°, centered at 2θ = 24.5° (*d* = 3.6 Å). The XRD of the PPA1 sample also showed peaks in the same angular region as that of PPA16; however, they were much more defined, as can be observed from Figure 1b. Around the peak centered at 2θ = 18.5° in PPA16, two sharp peaks for PPA1 appeared—one at 2θ = 17.8° (*d* = 5.0 Å) and another at 2θ = 18.6° (*d* = 4.8 Å). On the other hand, at around 2θ = 24.5° (centered peak of PPA16), PPA1 presented two well-defined peaks at 2θ = 23.8° (*d* = 3.7 Å) and 25.8° (*d* = 3.4 Å). Our results clearly showed an improved crystallinity in the PPA1 sample as a consequence of a synthesis condition such as the reagent concentrations.

We found a lack of structural reports on PPA in the literature. Hybrid materials based on PPA and clay were reported [21]. The formation of nanomaterials was confirmed by XRD results that demonstrated the intercalation of PPA into the clay phase by accessing increased interlayer spacing. The semicrystalline XRD patterns of the pure PPA presented peaks at 2θ = 3.58°, 7.38°, and 24.35°, which were significantly different from those presented in our study. The authors used HClO_4_ as dopant acid, which may have resulted in a different PPA crystal structure. The nature of the dopant acids significantly influenced the polymerization and crystallinity of conjugated polymers [2]. Moreover, the XRD pattern of polymeric blends of PPA and ZnO nanoparticles was also reported [17]. Despite presenting an XRD pattern similar to that of PPA16, the authors did not provide the angular positions of the pure PPA peaks. In addition, the XRD measurements started at 2θ = 10°, preventing the confirmation of the intense and narrow peak observed here at 2θ = 5.2°.

### 3.2. Morphological Evaluation

**Figure 2:**
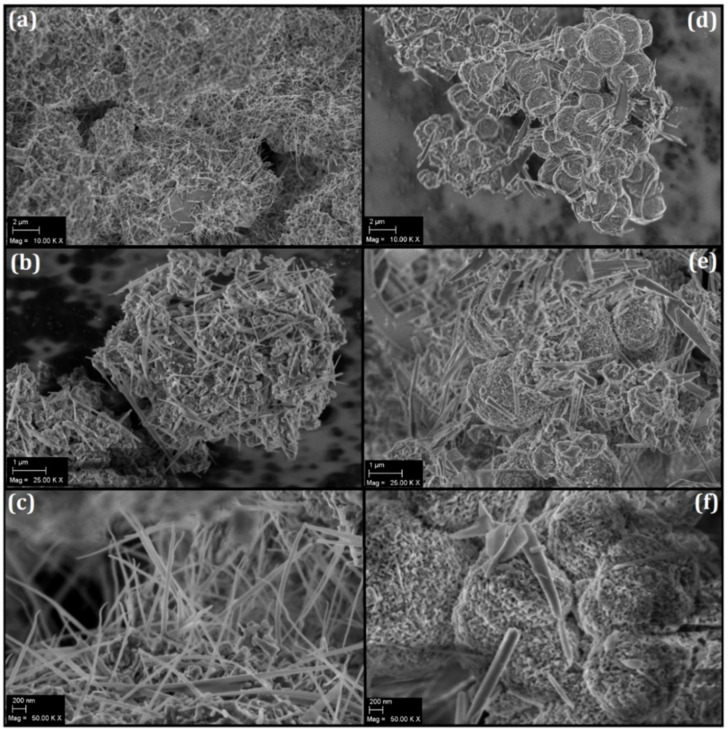
SEM images of (**a**–**c**) PPA1_HT_ and (**d**–**f**) PPA16_HT-HH_.

Scanning Probe Microscopy (SPM) techniques such as Scanning Tunneling Microscopy (STM), Atomic Force Microscopy (AFM), and even profilometry are based on the three-dimensional images of surfaces that are suitable for the study of fractal properties. However, the images produced by SEM have also been widely used in fractal and multifractal analyses of surfaces [58], providing two-dimensional images without information on the height profile. In this case, the SEM image of a fractal surface is not self-similar in all spatial directions and presents the advantage of not introducing the tip convolution effect (usually causing a systematic error in the estimative of the fractal dimension) [59]. Figure 2 shows the SEM images of (a–c) PPA1_HT_ and (d–f) PPA16_HT-HH_.

Basically, two types of morphology were observed in both samples: needles and globular particles. As previously observed in the XRD results, the diffractograms were typical of semicrystalline materials and narrow peaks were observed superimposed on a diffuse pattern. The SEM images suggested that the needle phase may have been related to the crystalline contribution of the polymer. These data became more consistent because the number of needles decreased in the PPA16_HT-HH_ sample, which also presented a lower percentage of crystallinity. On the other hand, according to the SEM images, the decrease in the percentage of crystallinity was accompanied by an increase in the globular morphology.

**Figure 3:**
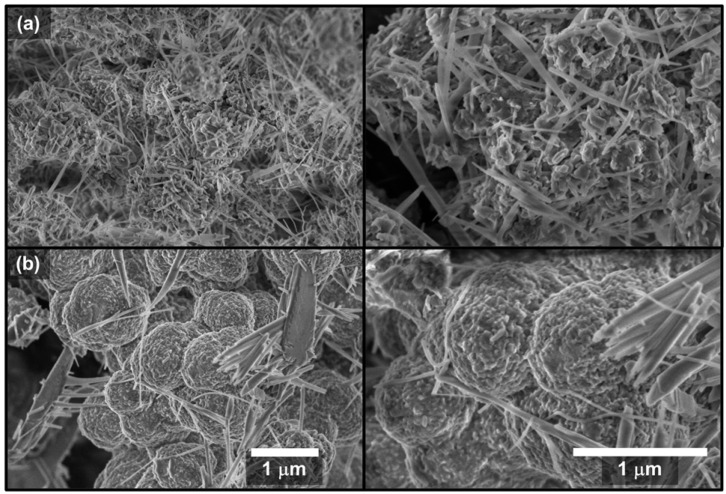
SEM micrographs of (**a**) PPA1_HT_ and (**b**) PPA16_HH-HH_. Magnification of 25,000× and 50,000×, respectively.

The SEM technique has the main advantage of generating images with a high pixel density due to its high resolution of 1024 × 768 pixels. Figure 3 shows the SEM micrographs (with magnifications of 25,000× and 50,000×) of PPA highlighting the influence of the synthesis parameters and concentration on the polymer’s morphology.

Figure 3a shows that the polymer surface is basically formed by elongated needles (up to a few microns in length), pointing toward a polymer growth direction. At a higher magnification (Figure 3a, right) and after applying a lighter-contrast image, some roughness was also observed. On the other hand, Figure 3b demonstrates a significant change in morphology when compared to that of Figure 3a. The elongated needles were present in a reduced amount while the globular morphology increased considerably and presented larger globules. In addition, the needles seemed to be wider and shorter.

**Figure 4:**
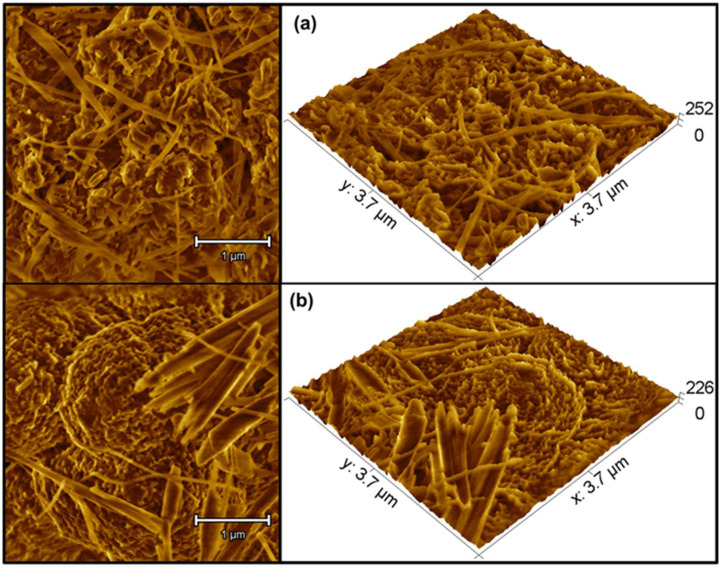
The 2D and 3D zoom reconstructions of SEM images (50,000×) for (**a**) PPA1_HT_ and (**b**) PPA16_Ht-HH_.

The SEM images are provided in grayscale to allow for a good indirect method by using a color gradient across the entire image. In this case, the black color represents the lowered area, the white color indicates the raised area, and the gray color (in various intensity levels) represents the height between the lowered and raised levels. Therefore, the relative elevation value of each point in the range of 0 to 255 for each gray value at each point in the SEM image was obtained [60]. For this reason, the images in Figure 3b (magnifications at 50,000×) were enlarged in specific regions using the software Gwyddion 2.59 [61]. Thus, Figure 4a,b show the 2D and 3D reconstructions of 3.7 mm × 3.7 mm square areas in which colors were used to highlight the differences between both samples. The *z*-axis of the 3D images does not have a metric dimension and only denotes the intensity variation in the gray levels of the original SEM images. These images were used for the multifractal study presented herein.

#### Multifractal Analysis

Digitized images (such as those provided by the SEM technique) can be used to obtain relevant quantitative surface information of a wide range of micro- and nanostructures through fractal analysis. A fractal dimension D (for 3D objects, 2 ≤ D ≤ 3) presenting higher values indicates substantial geometric details and irregularities. The multifractal analysis performed in this work represented a generalization of the fractal approach and could provide a more comprehensive description of the fractal surfaces [62].

Multifractal analysis describes the local behavior of measurements or functions in a geometrical and statistical method. Using the classical formalism of multifractal analysis, a spectrum of fractal dimensions (multifractal spectrum) can be obtained. Multifractality is used in the description of heterogeneous systems consisting of subsets that exhibit local self-similar properties as based on the concept of self-similarity, which requires the introduction of probability measurements [63].

**Figure 5:**
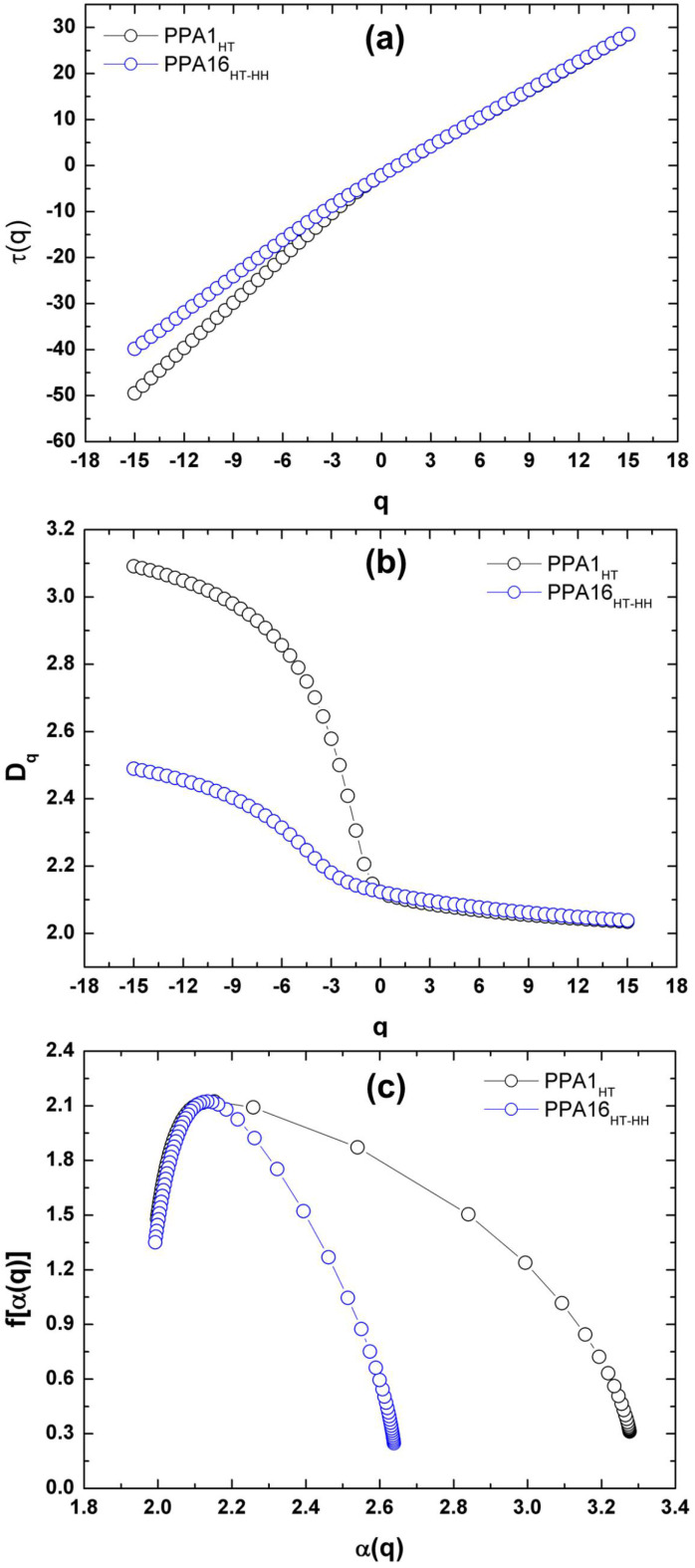
(**a**) Mass exponent τ(*q*), (**b**) generalized dimensions Dq, and (**c**) multifractal spectra (*f*(*α*) *versus α*) as a function of the order of moments for PPA1_HT_ and PPA16_HT-HH_.

Figure 5 shows the results obtained from the multifractal analysis of the images in Figure 4. The mass exponent (*τ*) as a function of the moment of order (*q*) (Figure 5a) indicated that both PPA1_HT_ and PPA16_HT-HH_ presented a nonlinear tendency, as well as a multifractal behavior that was more evident in PPA1_HT_.

The evidence of multifractality was supported by the nonconstant behavior of Dq versus *q* (Figure 5b) as well as by the concave curve of the multifractal spectrum *f(α)* versus *α* shown in Figure 5c. The *τ* and Dq parameters were calculated for different moment values (*q*) in a range of −15 < *q* < 15. As can observed in Figure 5, PPA1_HT_ clearly exhibited distinct characteristics that were confirmed by the estimated parameters related to the multifractal spectra presented in Table 2.

The parameter *Δf* represents the difference of fractal dimensions between the maximum and minimum singularity strength being calculated as *Δf* = *f*(*α_min_*) − *f*(*α_max_*), quantifying, in this way, the strength of the multifractality [64]. Thus, the analyzed surface is dominated by areas described by a high probability value when *Δf* > 0. However, when *Δf* < 0, the dominant areas are described by their low probability value. If *Δf* is significantly small, the height distribution of the mass deposited at the highest site is equal to that at the lowest sites, indicating more homogeneous structures [65]. On the other hand, a greater variation in *f* indicates a greater heterogeneity of the analyzed structure [66].

Figure 5c shows the shape and extent of the multifractal spectrum *f*(*a*) versus *a*, where the asymmetry of the distribution indicates the presence of multifractal. According to Table 2, in both PPA1_HT_ and PPA16_HT-HH_, all the *Df* values were similarly positive and higher for PPA1_HT_. The singularity spectrum was wider for PPA1_HT_ due to a greater surface heterogeneity and percentage of crystallinity (as previously observed in the XRD results). In addition, the spectrum was shifted to higher values and the multifractal spectrum of PPA1_HT_ showed a greater inclination to the right when compared to that of PPA16_HT-HH_. Our results showed that the surface became more irregular and complex when the crystallinity was reduced. As a consequence, higher values for the multifractality parameter *Δa* (*Δα* = *α_max_* − *α_min_*) were observed while the *Δf*(*a*) values were reduced. It can be seen in Figure 5c and Table 2 that PPA1_HT_ presented the greatest *Δα* width spectrum, pointing to the highest degree of multifractality.

Systematic structural studies have been performed using the AFM technique to describe the statistical parameters used to evaluate the complexity of an individual surface [67,68]. This technique allows for the understanding of the influence of the material surface on specific properties. As a result, statistical parameters related to the particle surfaces such as roughness, peak distribution, height distribution, and nanotexture homogeneity have been determined. A previous report [67] presented advanced morphological and fractal aspects of a polymeric particle surface containing an encapsulated essential oil that were evaluated using AFM topographical images. The authors pointed to the influence of the essential oil concentration on the particles’ morphology and surface roughness. This tool can also be useful in evaluating the quality standard in the development of novel materials for the controlled release of bioactive compounds.

A 3D nanoscale morphological surface analysis of polymeric particles containing different concentrations of bioactive compound was proposed elsewhere [68]. The authors verified that higher concentrations of a bioactive compound promoted a decrease in the dominant spatial frequencies of the particle surfaces. The proposed evaluation allowed access to stereometric parameters, which can be a guide in the development of novel particle carriers with desirable surface properties for technological applications based on microtexture roughness.

Fractal dimension calculations have also been performed for conjugated polymers [8]. A poly(*o*-methoxyaniline) emeraldine-salt form (ES–POMA) was subjected to a heat-treatment process, promoting a progressive reduction in crystallinity. SEM images were obtained to show the micromorphology changes induced by heating, resulting in a loss in globular morphology. Parameters based on statistical data that allowed characterization of the morphology and geometric structure were accessed. The untreated ES-POMA presented a greater distribution of heights; however, the heat-treated polymers exhibited a considerably reduced symmetrical behavior. The authors verified that the polymer presented a significant morphological change after the heating process (also based on Df values), pointing to smoother surfaces with smaller height variations.

### 3.3. ^13^C NMR Spectroscopy

The isomeric positions of anisidines (*meta*, *ortho,* or *para*) result in different ^13^C NMR spectra because the chemical environments of the carbon atoms (arrangements of neighboring nuclei) are also different [69]. These spectra are useful in estimating the positions of the methoxy and amine groups, as well as other carbon atom positions.

**Figure 6:**
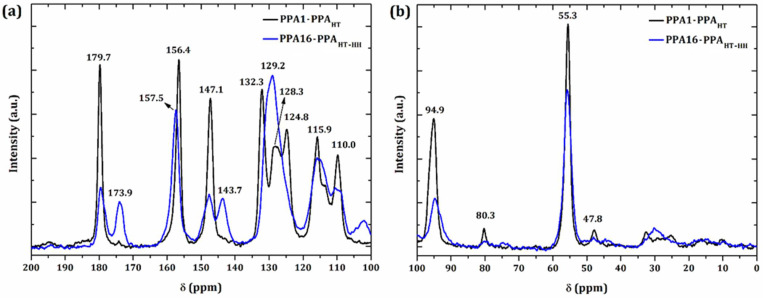
^13^C NMR spectra of PPA1_HT_ and PPA16_HT-HH_: (**a**) δ = (200–100) ppm; (**b**) δ = (100–0) ppm.

Figure 6 shows the non-normalized high-resolution solid-state ^13^C NMR spectra of PPA1 and PPA16. The ^13^C NMR spectra observed in a solid state, even when using magic-angle spinning (MAS), showed broader signals than the ^13^C NMR spectra recorded in solution due to the residual anisotropic effects (dipolar coupling) in response to the conformational changes, which regarded the differences in intramolecular interactions. The chemical shift signals between δ = (135–120) ppm observed in the PPA16 spectrum were more overlapped and less defined, suggesting a larger noncrystalline content. This result was previously observed in the XRD results.

The observed absence of signals centered at 173.9 ppm, 143.7 ppm, and 102.0 ppm in the PPA1 spectrum suggested a more regular crystal structure. In polymer materials, signals with a spectral profile similar to another next neighbor probably indicate a similar molecular structure, but in different positions or spatial arrangements. For this reason, our results suggested two different types of polymerizations: head-to-tail (HT) and head-to-head (HH) polymer chains.

The proposition of the molecular polymerization of PPA requires an understanding of the electron density distribution of aromatic rings. The methyl group was not allowed to participate in the polymerization process due to the mechanisms of proton loss/suppression or hydride migration. On the other hand, proton loss was highly improbable because the formed carbanion was significantly reactive. As the reaction started from the *p*-anisidine monomer, only the amine group and the *ortho* and *meta* positions could participate in the polymerization. The amino and carboxy groups are known to be a ring-activating groups, “increasing” the electron density at the respective *ortho* and *para* positions and “decreasing” the electron density of the respective *meta* positions.

This result indicated that the *ortho* and *para* positions became more electronically negative, while the opposite effect was expected for the *meta* position. However, due to the molecular symmetry, the *ortho* position of one group was the *meta* position of the other group. In this case, the amine group was more activating than the methoxy group, so the *meta* position related to the amine was “more positive” than that of the *meta* position related to the methoxy group. Similarly, the *ortho* position related to the amine group was “more negative” than that of methoxy group.

Solid-state polymerizations can generally be regarded as phase transitions from the crystalline phase of the monomer to the polymer growth phase. The character of these phase transitions determines the mechanism of the polymerization. It is therefore necessary to know the mechanism of the 3D order of the crystalline monomer transferred to the resulting polymer phase [43]. Our results suggested that the polymerization reaction started from both the nonbonding electrons of the amine groups and the oxygen atom. Thus, the bonds between these groups were not considered because the N–O, O–O, and N–N bonds were extremely unstable and photosensitive. The oxygen atoms did not find a more energetically stable situation for polymerization. What remained, in fact, was the bond between the amine groups and the carbon atoms in the *ortho* and *meta* positions. At this point, there were two possible considerations: (*i*) in the first situation (head-to-tail), the amine group was bonded at the *meta*-position of the amine from the neighboring residue through its nonbonding electrons. This polymerization pathway was considerably relevant because this carbon atom was the “most positive” of the aromatic ring; and (*ii*) as both carbon atoms (*ortho* and *meta*) were influenced by the amine and methoxy groups, the bond with other carbon atoms could also occur but to a lesser extent, resulting in a head-to-head molecular structure.

The morphology and texture of a solid-state polymer results from an overall reaction of the primary molecular structure. Thus, the knowledge of the crystal structure of the monomer, as well as the molecular structure and morphology of the polymer, is important in explaining the solid-state polymerization mechanism [43]. A detailed picture of the reaction mechanism of PPA is still very difficult to develop. However, the assignment of the solid-state ^13^C NMR signals of the carbon atoms of the PPA_HT_ and PPA_HH_ molecular structures, as based on the concept of electronic shielding, was useful. The electronic cloud field was vectorially opposed to that applied by the equipment. The denser this cloud (greater number of electrons), the greater the shielding from the external field. A high electron density usually means a greater polarization of the nuclei and lower upfield-shifted frequencies. However, the opposite situation was also considered, resulting in signals with further downfield-shifted frequencies. Therefore, the signals from the methoxy carbon were intense, highly polarized, and located far to the right of the spectrum.

The signals at 94.9 ppm and 102.0 ppm were assigned to an isolated carbon between the functional groups of the molecular structure. As mentioned previously, the amine group activated the *ortho* position, so two amines still activated more than one amine and one methoxy group. For this reason, a signal separation (94.9 ppm and 102.0 ppm) was observed that suggested that the head-to-tail molecular structure was predominant in PPA1. Similar results were observed in the signals at 147.1 ppm and 143.7 ppm. In this case, as one amine was located at the *meta* position relative to another in a head-to-tail molecule, the carbon core was slightly deshielded when compared to the same core in the head-to-head molecule (in which the amine groups were located at the *ortho* position to each other). Further evidence of these molecular structures was related to the signals at 179.7 ppm and 173.9 ppm based on the same interpretation. However, these signals were not found in the monomer spectrum [69], indicating an effective polymerization/conjugation due to the presence of –N = bonds.

**Figure 7:**
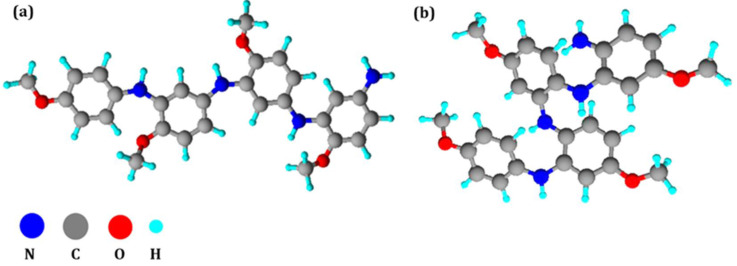
Proposed molecular structures of (**a**) PPA_HT_ and (**b**) PPA_HH_ according to the ^13^C NMR results.

The proposed molecular structures of PPA_HT_ and PPA_HH_ are shown in Figure 7. Furthermore, the spectra signals were useful in estimating the percentage of head-to-tail and head-to-head molecular structures: PPA1 is fully (100%) constituted of head-to-tail polymer chains (PPA_HT_), while PPA16 is formed predominantly by a head-to-tail molecular structure ((62.0 ± 0.5)%; PPA_HT-HH_)).

The structure of a polymer network is normally determined by polymer chains of different lengths that are disordered in a random orientation [70]. Our results suggested that the homogeneity of the head-to-tail polymerization of PPA resulted in an enhanced percentage of crystallinity, as shown by the XRD and ^13^C NMR results. A combination of head-to-tail and head-to-head polymer chains influenced the amount of the noncrystalline phase. On the other hand, the concentration of *p*-anisidine monomer, APS and, HCl also affected the mechanism of polymerization; their lower concentration favored a more crystalline and homogeneous head-to-tail polymerization.

### 3.4. Geometry Optimization

A quantum-chemical investigation of the molecular structure of PPA was carried out using the geometry optimization of the head-to-tail (PPA_HT_) and head-to-head (PPA_HH_) tetramers in the ground state.

**Figure 8:**
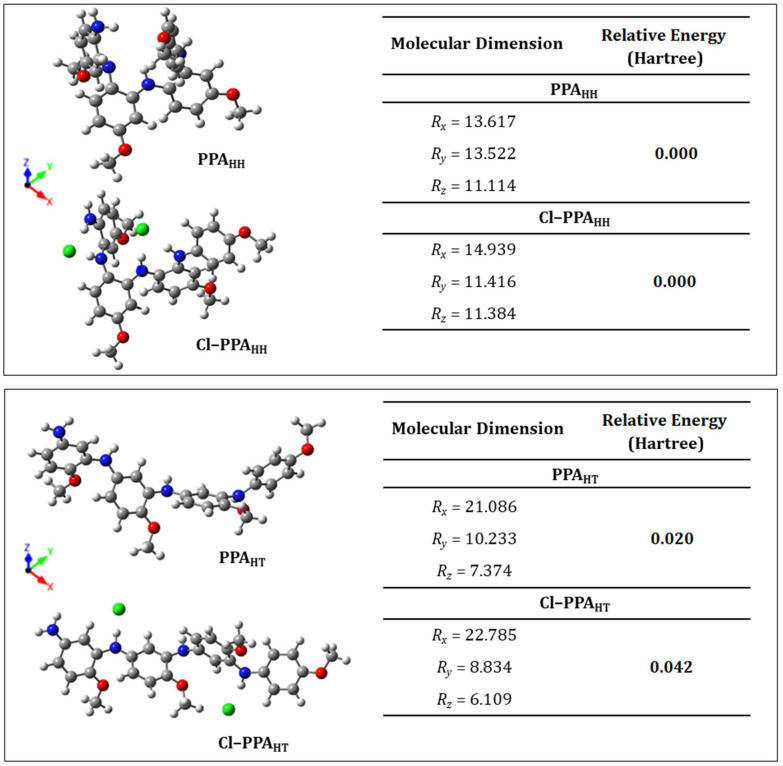
PPA_HT_, Cl–PPA_HT_, PPA_HH_, and Cl–PPA_HH_ tetramers and their respective molecular dimensions and energy values after relaxation.

An optimization also was performed for the molecular structures of the chlorine-doped PPA. The Cl^−^ counterion was incorporated into the molecular structures of PPA_HT_ and PPA_HH_ to obtain the doped polymers Cl–PPA_HT_ and Cl–PPA_HH_, respectively. Figure 8 shows the PPA_HT_, Cl–PPA_HT_, PPA_HH_, and Cl–PPA_HH_ tetramers and their respective molecular dimensions and energy values. The molecular structures of the doped tetramers presented different dimensions: the *x*-dimension increased, the *y*-axis was reduced, and the *z*-lattice presented a marginal modification.

Polyaniline and its derivatives can present some degree of molecular organization. However, the doping process influences the arrangement of the polymer chains [4]. The doped poly(*o*-methoxyaniline) presented a percentage of crystallinity ranging from 48 to 63% as a function of the time of polymerization. However, after the neutralization process to obtain its undoped form, the crystallinity was reduced to 27%, showing the influence of the counterions on the molecular chains’ alignment [14,71]. Charged nanoparticles can also influence the alignment of conducting polymer chains [72]: gold nanoparticles stabilized with sodium citrate were able to form a complex with a polyaniline emeraldine salt form, considerably reducing the percentage of crystallinity of the resulting material.

The doped conducting polymers usually presented a higher percentage of crystallinity and electrical conductivity. Furthermore, the head-to-tail and head-to-head polymerization (or their combination as observed in PPA16_HT-HH_) could result in particular electronic properties controlled both by bond length and torsional angle dimerization, since the phenylene rings moved from the plane defined by the nitrogen atoms to reduce the strong sterical hindrance [73]. As shown in Figure 8, these torsions were considerably different among the PPA_HT_, Cl–PPA_HT_, PPA_HH_, and Cl–PPA_HH_ structures and could influence the delocalization and mobility of the charge carriers.

**Figure 9:**
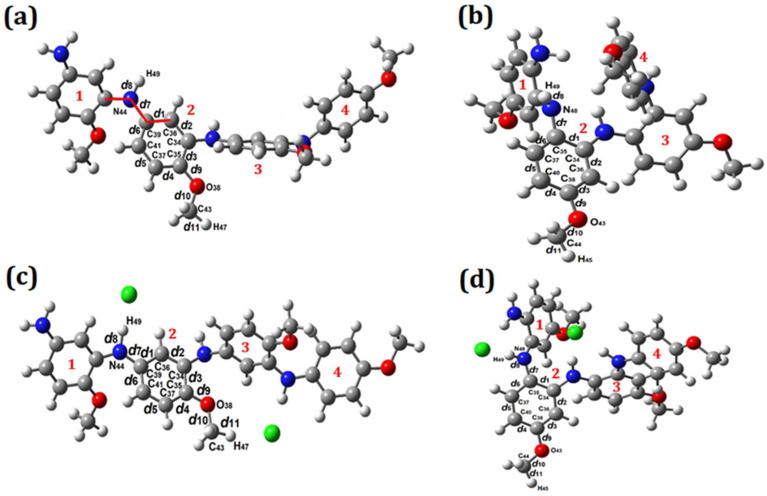
Geometric optimization of PPA_HT_, Cl–PPA_HT_, PPA_HH_, and Cl–PPA_HH_ tetramers: (**a**) undoped PPA_HT_; (**b**) undoped PPA_HH_; (**c**) doped Cl–PPA_HT_; (**d**) doped Cl–PPA_HH_. Interatomic distances are identified as *d*_1_ to *d*_11_. Numbers 1–4 represent the repeated unit from each polymer.

After evaluating the influence of the counterions on the molecular dimensions and energy values of the PPA_HT_, Cl–PPA_HT_, PPA_HH_, and Cl–PPA_HH_ tetramers, a repeated unit from each polymer was structurally characterized as shown in Figure 9a–d with the interatomic distances labeled as *d_n_* (n=1−11).

Table 3 shows the interatomic distances from *d*_1_ to *d*_11_ highlighted in Figure 9. Despite presenting two possible mechanisms of polymerization (head-to-tail and head-to-head), the resulting polymer molecules (PPA_HT_ and PPA_HH_) did not present significant differences in their interatomic distances and angle values. The angles at the carbon-ring-bonded nitrogen atoms were almost similar for both types of polymerizations (for PPA_HT_: *d*_2_↔*d*_3_ = 30.881° and *d*_1_↔*d*_6_ = 30.881°; for PPA_HH_: *d*_1_↔*d*_2_ = 30.386° and *d*_1_↔*d*_6_ = 30.695°), revealing that the phenyl ring torsions were not considerably influenced by the type of polymerization.

The doped tetramers (Cl–PPA_HT_ and Cl–PPA_HH_) showed small variations in their interatomic distances and angle values in the planes of the highlighted monomers, showing that the doping process did not cause significant structural changes. However, the *d*_8_ (N−H bond) values of both Cl–PPA_HT_ and Cl–PPA_HH_ increased from 1.016 Å to 1.101 Å and from 1.023 Å to 1.076 Å, respectively. These appreciable increments in the N–H interatomic distances revealed a local influence of the counterions on the molecular structures that might have been the protonation of the hydrogen atom. The protonation process preferentially occurred at the imine nitrogen atoms followed by an internal redox reaction, resulting in a semiquinone segment [74,75]. The doped forms of PPA revealed the influence of doping on the molecular structure related to the typical protonation process of polyaniline and its derivatives. The conduction mechanism of the salt form of polyanilines (doped forms) allowed the generation and disappearance of charged sites, while electroneutrality was maintained by mobile counterions [76]. Indeed, the physicochemical properties of these macromolecules also depended on the counterion of the Bronsted doping acid. Although counterions were needed for the compensation of charge, their nature and size significantly influenced the electrical conductivity of the conjugated polymers [2]. Finally, the results for the interatomic distances and angle values showed that both polymerization mechanisms (head-to-tail and head-to-head) were possible and depended on the synthesis parameters. In addition, structural characteristics were maintained after polymerization and the major influence on the molecular packing was performed by the counterions in the doped polymers.

The presence of counterions allowed for a greater regularity of the polymer tetramer when compared to the undoped systems. Differences were observed between the dihedral angles measured between two rings (as highlighted in red in Figure 9) when comparing the doped systems with their respective undoped systems. The angles between the rings 1–2, 2–3, and 3–4 of the PPA_HT_ structures (Figure 9a,c) changed by 6.70°, 65.13°, and 10.07°, respectively. The largest difference was found in the angle related to the 2–3 rings, which was the region that presented the greatest interaction between the counterions and polymer chain. The absence of counterion–chain interactions in the undoped system increased the freedom of rotation of the repetitive unit, twisting the chain in a shape tending toward a spherical conformation.

When considering the PPA_HH_ structures (Figure 9b,d), the greatest difference in the dihedral angles was observed in relation to the end of the polymeric chain. The rings 1–2 and 3–4 showed differences of 95.35° and 101.35°, respectively, when the doped systems were compared to their respective undoped ones. The rotation of ring 1 with respect to ring 2 was clearly observed after doping, as well as the rotation of ring 4 with respect to ring 3. For this reason, we concluded that counterions, in addition to modifying the electronic structure, directly influenced the structural configuration of the polymer chains.

### 3.5. FTIR Analysis

**Figure 10:**
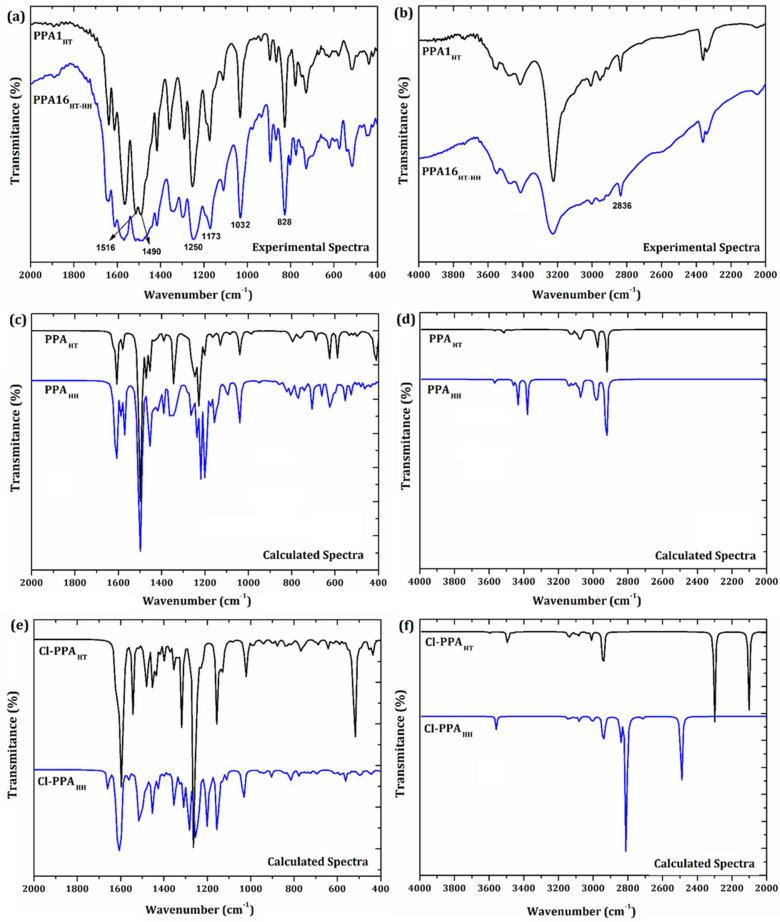
Experimental FTIR spectra of PPA1_HT_ and PPA16_HT-HH_: (**a**) from 2000 cm^−1^ to 400 cm^−1^ and (**b**) from 4000 cm^−1^ to 2000 cm^−1^; calculated FTIR spectra of PPA_HT_ and PPA_HH_: (**c**) from 2000 cm^−1^ to 400 cm^−1^ and (**d**) from 4000 cm^−1^ to 2000 cm^−1^; and calculated spectra of Cl–PPA_HT_ and Cl–PPA_HH_: (**e**) from 4000 cm^−1^ to 2000 cm^−1^ and (**f**) from 4000 cm^−1^ to 2000 cm^−1^.

The FTIR spectra of the PPAs were analyzed by considering the correlation between the data obtained experimentally for PPA1_HT_ and PPA16_HT-HH_ and those calculated for PPA_HT_ and PPA_HH_. Figure 10a–d shows the experimental (PPA1_HT_ and PPA16_HT-HH_) and calculated (PPA_HT_ and PPA_HH_) FTIR spectra, highlighting the main absorption bands.

The bands located from 2905 cm^−1^ to 3006 cm^−1^ (Figure 10b) in the experimental spectra of PPA1_HT_ and PPA16_HT-HH_ were assigned to *ν*(N–H). Similar bands were identified from 3515 cm^−1^ to 3518 cm^−1^ and from 3427cm^−1^ to 3514 cm^−1^ in the theoretical spectra of PPA_HT_ and PPA_HH_, respectively. The aromatic *ν*(C–H)*_ring_* bands were observed from 3080 cm^−1^ to 3138 cm^−1^ and from 3085 cm^−1^ to 3159 cm^−1^ in the calculated spectra of PPA_HT_ and PPA_HH_, respectively. A broad band assigned to *ν*(C–H)*_ring_* was found at 2836 cm^−1^ in the experimental spectra of PPA1_HT_ and PPA16_HT-HH_. Similar experimental results were previously reported [17].

The absorption bands located at 1242 cm^−1^ (PPA_HT_) and 1249 cm^−1^ (PPA_HH_) in the calculated spectra were related to *ν*(C–O–CH_3_). Correlated absorption bands were found at 1250 cm^−1^ in the experimental spectra of PPA1_HT_ and PPA16_HT-HH_.

The band located at 1230 cm^−1^ in the calculated spectra of PPA_HT_ and PPA_HH_ was assigned to *ν*(C–O). This band was found at 1173 cm^−1^ in the experimental spectra of PPA1_HT_ and PPA16_HT-HH_.

Bands related to *ν*(CH_3_) were observed in the experimental spectra of PPA1_HT_ and PPA16_HT-HH_ at 1418 cm^−1^ [20]. These bands were found at 1454 cm^−1^ in the calculated spectra.

The band related to the out-of-plane *γ*(C–H) in the aromatic rings was observed at 828 cm^−1^ in the PPA1_HT_ and PPA16_HT-HH_ spectra. Similar results were previously reported [21]. Correlated absorption bands were found in the theoretical spectra at 795 cm^−1^ and 774 cm^−1^ for PPA_HT_ and PPA_HH_, respectively.

The bands at 1216 cm^−1^ and 1228 cm^−1^ were found in the calculated spectra of PPA_HT_ and PPA_HH_, respectively, due to *ν*(C–N–C). Correlated bands presented a blueshift to 1109 cm^−1^ in the experimental spectra, probably as a consequence of the restrictions imposed by the coiled conformation of the bulk polymeric chains, as well as the combination of HT and HH polymerization.

Other absorption bands were observed due to *ν*(O–CH_3_) at 1039 cm^−1^ in the calculated spectra. Similar bands were verified in the experimental spectra at 1032 cm^−1^.

Bands observed in the experimental spectra of PPA1_HT_ and PPA16_HT-HH_ at 1490 cm^−1^ and 1516 cm^−1^ (Figure 10a) were assigned to the quinoid (Q) and benzenoid (B) structures, respectively [17]. However, these bands appeared to be less defined in the PPA16_HT-HH_ spectrum. These absorption bands were related to the doping level of polyaniline and its derivatives [4]. The bands assigned to the benzenoid structure were found at 1606 cm^−1^ in the theoretical spectra of PPA_HT_ and PPA_HH_.

The Q and B structures present an important role in the oxidation states of polyaniline and its derivatives; both structures comprise the emeraldine salt form [77]. The ratio between the band areas of the quinoid and benzenoid structures (Q/B) is useful in estimating the doping level of polyaniline and its derivatives [78]. Significant modifications in the Q and B bands were observed in the PPA1_HT_ and PPA16_HT-HH_ spectra. The Q/B value was found to be 1.0 for PPA1_HT_ and 0.8 for PPA16_HT-HH_, indicating a decreased doping level in PPA16_HT-HH_. Moreover, PPA1_HT_ presented a similar amount of Q and B structures, as expected in a half-oxidized emeraldine salt form. This difference suggested that the Q structures were in smaller amount in the PPA16_HT-HH_ polymer chains, probably due to the lower degree of oxidation. This result could be correlated with the reagent concentration of PPA16_HT-HH_ synthesis, influencing the percentage of crystallinity as well as the molecular structure conformation (combined HT and HH mechanisms of polymerization).

The structure and morphology of the hydrochloride polyaniline emeraldine salt form (ES-PANI) as synthesized by conventional and interfacial polymerization were evaluated based on different doping acid concentrations [4]. The FTIR spectra were useful in evaluating significant changes in the Q and B bands: the conventional polymerization resulted in Q/B values from 0.4 to 0.6, indicating that the doping level increased for a higher dopant acid concentration. An even more intense dopant effect was verified in the polymers that resulted from interfacial polymerization, presenting Q/B values from 0.7 to 0.9. These results revealed the more efficient doping level as a result of the interfacial polymerization. The conventional and interfacial mechanisms of polymerization enhanced the percentage of crystallinity of polyaniline with different structures, suggesting that interfacial polymerization allowed for a better alignment of the polymer chains. Table 4 shows the main experimental and calculated absorption bands from the FTIR spectra of PPA while also considering the doped molecular structures.

Figure 10e–f shows the theoretical spectra of the Cl–PPA_HT_ and Cl–PPA_HH_ structures that resulted from the doping process in the polymer chains. The spectra show the *γ*(C–H) absorption in the aromatic rings at 763 cm^−1^ and 759 cm^−1^, respectively. The *ν*(O–CH_3_) band was found at 1024 cm^−1^ and 1032 cm^−1^ for Cl–PPA_HT_ and Cl–PPA_HH_, respectively. The *ν*(C–O–CH_3_) vibration was located at 1253 cm^−1^ (Cl–PPA_HT_) and 1257 cm^−1^ (Cl–PPA_HH_), respectively. The bands at 1223 cm^−1^ and 1235 cm^−1^ were assigned to *ν*(C–N–C) in the spectra of Cl–PPA_HT_ and Cl–PPA_HH_, respectively.

The bands related to the Q structure were located at 1537 cm^−1^ (Cl–PPA_HT_) and 1513 cm^−1^ (Cl–PPA_HH_). The B structure was responsible for the absorption bands at 1596 cm^−1^ (Cl–PPA_HT_) and 1602 cm^−1^ (Cl–PPA_HH_).

The *ν*(N–H) bands observed in the calculated PPA_HT_ and PPA_HH_ presented a significant blueshift in the spectra of the doped structures (Cl–PPA_HT_ and Cl–PPA_HH_) due to the influence of the counterions on the polymer chains. Additional intense bands were observed in the calculated spectra of Cl–PPA_HT_ at 2101 cm^−1^ and 2300 cm^−1^ (Figure 10f) that were assigned to *ν*(N–H) being influenced by the doping process. However, the *ν*(N–H) bands suffered a blueshift (including additional bands) in the Cl–PPA_HH_ spectra from 2491 cm^−1^ to 2841 cm^−1^. This was due to the expected electrostatic interaction between (H49) and counterions during the doping process, which influenced the N–H bond length/stretching absorption.

Despite being influenced by the presence of counterions, the HH and HT mechanisms of polymerization resulted in different molecular structures of PPA. In addition, the steric hindrance imposed by the head-to-head polymerization also may have contributed to impairing the N–H stretching. Moreover, the *ν*(N–H) bands were found in the range of 2905 cm^−1^–3006 cm^−1^ in the experimental spectra of both PPA1_HT_ and PPA16_HT-HH_ due to the same reasons described above, and also probably due to the larger concentration of head-to-tail polymerization in PPA16_HT-HH_ (as observed from the ^13^C NMR results: (62.0 ± 0.5) %).

In general, the main changes in the calculated spectra showed the influence of counterions on the molecular structure, especially on the *ν*(N–H) absorption. On the other hand, the experimental spectra did not show significant differences between PPA1_HT_ and PPA16_HT-HH_, probably due to the predominance of the head-to-tail polymerization in both structures. When comparing the calculated and experimental spectra, the most pronounced changes were observed in *ν*(C–H), *ν*(N–H), *ν*(C–O), and *ν*(C–N–C); these were caused by the doping process as well as the different mechanisms of polymer conformation.

### 3.6. Electrical Conductivity

The degree of protonation and conductivity have been found in a wide range in PANI and its derivatives, mainly due to differences in the conformation of the polymer chains and packing [79]. While the structure of the π-conjugated backbone is responsible for imparting the core optoelectronic and electrochemical functionality of the polymer, side chains appended to the backbone play an important role in tuning these properties [80].

The mechanisms of charge conduction are still not completely understood, mainly due to the diversity of factors that affect conductivity along the polymer chains in PANI and its derivatives. More specifically, side chains may influence the doping mechanism and efficiency, long-range order and polymer packing, and morphology of conjugated polymers [79].

**Figure 11:**
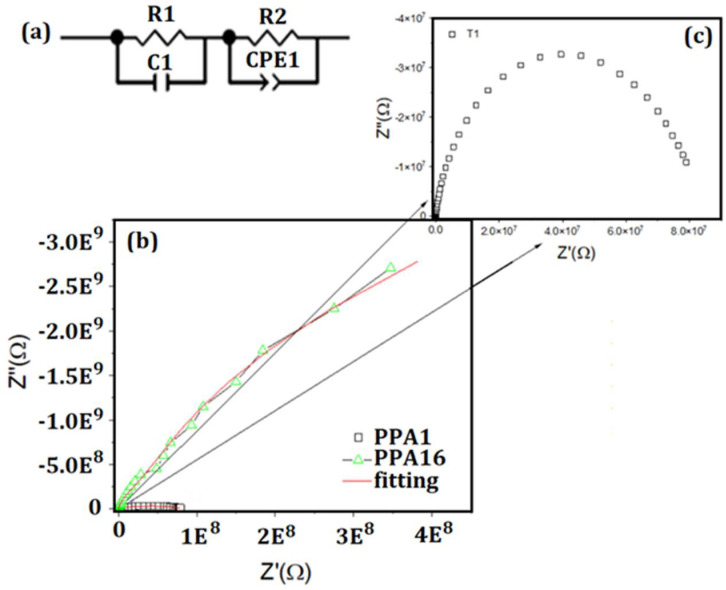
(**a**) Equivalent circuit used for sample adjustment; (**b**) Cole–Cole diagram with symbols for the different polymers; (**c**) enlargement of the Cole–Cole diagram of the PPA1_HT_ polymer. The solid red lines represent the adjustment by the equivalent circuit in the graph.

Analyses based on a combination of techniques including *dc* conductivity measurements can provide useful information on the nature of charge localization. The electrical conductivity of most PANI-based materials was found to show a temperature dependence [81]. Figure 11a represents the equivalent circuit used to adjust the polymers PPA1_HT_ and PPA16_HT-HH_, where R_1_ and R_2_ correspond to the resistance of the most crystalline region (considered here as conducting islands) and the region of lower crystallinity, respectively. The capacitance and the phase constant element are represented by C_1_ and CPE_1_, respectively. The values for CPE_1_ close to 1 corresponded to a more capacitive character, while values around 0.5 were associated with a more resistive material.

Figure 11b shows the Cole–Cole diagrams and their respective adjustments for PPA1_HT_ and PPA16_HT-HH_. In this case, the semicircle with a larger diameter (higher strength) corresponded to PPA16_HT-HH_, which possessed a lower crystallinity as previously revealed by the XRD results. In the results obtained by the equivalent circuit, the total resistance (*R_t_* = *R_1_* + *R_2_*) of this polymer was found to be around 10^10^ Ω. A reduced crystallinity may have negatively influenced the mobility of the charge carriers due to the lack of conducting paths/islands, allowing the long-range mobility. Finally, the total resistance of PPA1_HT_ (which showed the highest crystallinity) was found to be on the order of 10^7^ Ω. Figure 11c shows a better visualization of the Cole–Cole diagram for PPA1_HT_. Table 5 shows the *R_t_* and polymer resistivity (*ρ* = R_t_ × S/d) values, where *S* and *d* are the area and thickness of the pellets, respectively.

The capacitance values were similar and of the same order of magnitude in PPA1_HT_ and PPA16_HT-HH_. However, a significant difference in resistivity values was observed in the range of 8.23 × 10^2^ MΩcm and 2.56 × 10^7^ MΩcm for PPA1_HT_ and PPA16_HT-HH_, respectively. Considering the extreme points of PPA1_HT_ (maximum crystallinity) and PPA16_HT-HH_ (minimum crystallinity), a difference in resistivity of five orders of magnitude was observed.

It is widely accepted that the doped molecules of PANI and its derivatives are not uniformly distributed, but rather agglomerated into conducting islands [79]. Furthermore, as structural disorder is associated with the localization of charges within the polymer matrix, this leads to the development of electronic traps, which limits the charge transfer, a phenomenon known as charge transfer hindrance [82]. Most conducting polymers show irregular channels with small islands immersed in a less dense, insulating matrix. A previous report pointed to the increased number of conducting islands as a function of the degree of doping in POEA [79].

Our results indicated that the resistivity values of PPA1_HT_ and PPA16_HT-HH_ were closely related to the amount of conducting crystallites in a noncrystalline matrix. It is not known whether the morphologies previously observed in the SEM images were purely related to the crystalline (needles) and noncrystalline (globules) phases. A previous report on POMA [14] evaluated the morphology of the doping form as entirely constituted of vesicular globules that presented a considerable degree of crystallinity as a function of the time of synthesis. For this reason, the globules, which were better observed in PPA16_HT-HH_, may also have presented a semicrystalline structure.

We can state that the synthesis conditions resulted in polymers with different structural characteristics and different levels of doping, which directly influenced the resistivity values. The chains in the crystalline regions (more pronounced in PPA1_HT_) should have been better aligned, which would increase the electron delocalization. There is a finite density of states of conduction electrons around the Fermi levels in doped conjugated polymers, and the carriers may be spatially localized due to the structural disorder [8]. In the case of PPA16_HT-HH_, the homogeneously disordered regions limited the overlapping of wave functions [42]. In this case, conduction could only take place through hopping [79,83]. This result may have been a consequence of the nonexistence of efficient conducting paths allowing the long-range mobility of charge carriers. The resistivity values obtained in this work, as reported in Table 5, were similar to those previously reported [84].

A wide range of electrical conductivity values were found in the scientific literature for *ortho*, *meta,* and *para*-substituted polyanilines. The emeraldine salt form of POMA was chemically synthesized with a time of synthesis ranging from 0.5 to 72 h [14]. The authors observed that the percentage of crystallinity increased as a function of time and that the polymer obtained at 72 h presented the highest value for electrical conductivity. Interestingly, the more conductive crystalline POMA presented an undefined morphology and showed a loss in globular morphology when the time of synthesis was increased. The electrical conductivity of POMA was found to be 5.18 × 10^−7^ S/cm (0.5 h of synthesis, 48% crystallinity) to 8.89 × 10^−7^ S/cm (72 h of synthesis, 63% crystallinity).

The electrical conductivity of poly(*m*-anisidine) (PMA) as a function of frequency and different dopant acids was found to be 3.32 × 10^−7^ S/cm, 5.16 × 10^−9^ S/cm, and 4.95 × 10^−10^ S/cm for PMA/H_2_SO_4_, PMA/HNO_3_, and PMA/HCl, respectively [2]. These values were similar to those previously calculated from the total resistance. The nature of the doping acid and counterion size significantly influenced the charge mobility in the conjugated polymers. However, the authors observed that the PMA doped with HCl surprisingly presented a reduced electrical conductivity. A theoretical evaluation via DFT was performed that verified a charge transfer between the polymer chains and counterions. The PMA/HCl polymer presented the HOMO band partially filled, while in PMA/H_2_SO_4_, a fully occupied band was verified, resulting in a near-zero-gap semiconductor behavior.

There is, however, a lack of consistent data on the electrical conductivity of PPA. Moreover, the few published results that were available in the literature varied over a wide range of values, making it difficult to find a correlation among them. Composites formed by PPA and MnO_2_ were prepared in different concentrations via oxidative polymerization using HCl as dopant acid and KIO_3_ as an oxidizing agent [85]. The authors reported the electrical conductivity of the pure PPA in a range of 5.9 × 10^−4^ S/cm; the PPA/MnO_2_ composites presented a reduced electrical conductivity when the percentage of MnO_2_ was increased from 8.6 × 10^−3^ S/cm (13 % of MnO_2_) to 5.2 × 10^−3^ S/cm (52 % MnO_2_). Another published study proposed the preparation of nanocomposites synthesized using oxidative polymerization of *p*-anisidine and/or aniline monomers with TiO_2_ nanoparticles in the presence of hydrochloric acid and ammonium persulfate. The electrical conductivity values of the nanocomposites were found in the range of 0.08–0.91 S/cm, following the tendency of the pure polymers [20]. The composite based on PANI and PPA presented higher and lower electrical conductivity values, respectively. The pure PPA presented an electrical conductivity of 0.22 S/cm.

## 4. Conclusions

Poly(*p*-anisidine) was successfully synthesized based on different concentrations of the monomer, dopant acid, and oxidizing agent, resulting in polymers with different percentages of crystallinity. Their structural, morphological, spectroscopic, and electrical properties were found to be significantly related to the nature of the monomer polymerization.

The major challenge of this research was to understand and propose a bonding mechanism of the *p*-anisidine monomers to form the PPA polymer chains. The ^13^C NMR analysis allowed for the proposition of the polymerization mechanisms, revealing that PPA1_HT_ was purely formed by head-to-tail (HT) polymerization, while PPA16_HT-HH_ was formed by two different molecular arrangements composed of head-to-tail (HT) and head-to-head (HH) polymerization. The scientific literature was not clear about the fashion in which the *p*-anisidine monomers formed the polymeric structure. Then, the HT and HH mechanisms of polymerization resulted in different crystal structures and morphologies that were correlated to explain the SEM images and XRD patterns. Basically, the needle-like morphology mainly resulted from the HT polymerization while a mixture of needles and globules essentially formed the morphology of the combined HT-HH polymerization.

Most of experimental results were supported by theoretical analysis via DFT, confirming the fashion in which the polymer chains were formed in both PPAs. These results were significantly important for spectroscopic evaluation, allowing the confirmation of the main vibrational stretching modes in both HT and HH polymers. The calculated spectra pointed to the influence of counterions on the molecular structure, especially on the *ν*(N–H) absorption. On the other hand, the experimental spectra did not show significant differences between PPA1_HT_ and PPA16_HT-HH_, probably due to the predominance of the head-to-tail polymerization in both structures. Finally, the electrical conductivity results revealed the resistive behavior of the as-synthesized PPA.

All results proposed herein on PPA are considerably important to the scientific community due to the lack of information on *para*-substituted polyanilines. We hope this research can stimulate further studies on PPA, since other information (such as thermal and optical properties) is still necessary. Due to the important role of conjugated polymers in novel technological applications, we highlighted the importance of the combined experimental and theoretical results of this study for a better understanding of the experimental results. Finally, the possibilities of research on PANI, POMA, PMA, and PPA continue to be extremely broad, promising, and capable of competing directly with other semiconducting materials.

## Figures and Tables

**Table 1:** Parameters of the syntheses of PPA based on the CCRD method.

PPA	*p*-Anisidine (g)	APS (g)	HCl (*M*)
PPA1	2.0	4.0	1.0
PPA2	2.0	8.0	2.0
PPA3	7.0	4.0	2.0
PPA4	7.0	8.0	1.0
PPA5	4.5	6.0	1.0
PPA6	2.0	4.0	2.0
PPA7	2.0	8.0	1.0
PPA8	7.0	4.0	1.0
PPA9	7.0	8.0	2.0
PPA10	4.5	6.0	1.5
PPA11	0.3	6.0	1.5
PPA12	8.7	6.0	1.5
PPA13	4.5	2.6	1.5
PPA14	4.5	9.3	1.5
PPA15	4.5	6.0	0.7
PPA16	4.5	6.0	2.3
PPA17	4.5	6.0	1.5

**Table 2:** Parameters of the multifractal spectra.

Parameters	PPA1_HT_	PPA16_HT-HH_
*f*(*α_max_*)	0.34	0.14
*f*(*α_min_*)	1.49	1.34
*Δf* = *f*(*α_min_*) − *f*(*α_max_*)	1.15	1.20
*α* *_max_*	3.24	2.75
*α* *_min_*	2.04	2.04
*Δα* = *α_max_* − *α_min_*	1.20	0.71

**Table 3:** Interatomic distances (Å) and angle values observed in PPA_HT_, Cl–PPA_HT_, PPA_HH_, and Cl–PPA_HH_ tetramers.

PPA_HT_		PPA_HH_	
Interatomic Distances (Å)	Angles (°)	Interatomic Distances (Å)	Angles (°)
*d*_1_ = 1.407	(N_44_ C_39_ C_36_) = 118.280	*d_1_* = 1.431	(N_48_ C_35_ C_34_) = 118.103
*d*_2_ = 1.400	(C_35_ O_38_ C_43_) = 117.437	*d_2_* = 1.403	(C_38_ O_43_ C_44_) = 117.364
*d*_3_ = 1.421	(C_36_ C_39_ C_34_) = 28.816	*d*_3_ = 1.405	(C_34_ C_35_ C_36_) = 30.386
*d*_4_ = 1.399	(C_34_ C_36_ C_35_) = 30.881	*d*_4_ = 1.402	(C_36_ C_34_ C_38_) = 29.660
*d*_5_ = 1.400	(C_37_ C_41_ C_35_) = 29.240	*d*_5_ = 1.403	(C_40_ C_37_ C_38_) = 30.861
*d*_6_ = 1.401	(C_39_ C_41_ C_36_) = 30.881	*d*_6_ = 1.392	(C_35_ C_37_ C_34_) = 30.695
*d*_7_ = 1.409		*d*_7_ = 1.427	
*d*_8_ = 1.016		*d*_8_ = 1.023	
*d*_9_ = 1.371		*d*_9_ = 1.372	
*d*_10_ = 1.426		*d*_10_ = 1.424	
*d*_11_ = 1.097		*d*_11_ = 1.097	
**Cl–PPA_HT_**		**Cl–PPA_HH_**	
**Interatomic** **Distances (Å)**	**Angles (°)**	**Interatomic** **Distances (Å)**	**Angles (°)**
*d*_1_ = 1.419	(N_44_ C_39_ C_36_) = 116.773	*d*_1_ = 1.446	(N_48_ C_35_C_34_) = 125.903
*d*_2_ = 1.387	(C_35_ O_38_C_43_) = 120.253	*d*_2_ = 1.412	(C_38_ O_43_C_44_) = 118.142
*d*_3_ = 1.441	(C_36_ C_39_ C_34_) = 28.763	*d*_3_ = 1.392	(C_34_ C_35_ C_36_) = 30.563
*d*_4_ = 1.415	(C_34_ C_36_ C_35_) = 31.386	*d*_4_ = 1.419	(C_36_ C_34_ C_38_) = 29.012
*d*_5_ = 1.383	(C_37_ C_41_ C_35_) = 29.908	*d*_5_ = 1.379	(C_40_ C_37_ C_38_) = 30.869
*d*_6_ = 1.425	(C_39_ C_41_ C_36_) = 30.459	*d*_6_ = 1.420	(C_35_ C_37_ C_34_) = 31.088
*d*_7_ = 1.367		*d*_7_ = 1.382	
*d*_8_ = 1.101		*d*_8_ = 1.076	
*d*_9_ = 1.335		*d*_9_ = 1.362	
*d*_10_ = 1.445		*d*_10_ = 1.431	
*d*_11_ = 1.100		*d*_11_ = 1.096	

**Table 4:** Experimental and calculated absorption bands in the FTIR spectra of PPA_HT_, PPA_HH_, Cl–PPA_HT_, Cl–PPA_HH_, PPA1_HT_, and PPA16_HT-HH_.

	Theoretical Absorptions				Experimental Absorptions	
Absorption Bands	PPA_HT_ (cm^−1^)	PPA_HH_ (cm^−1^)	Cl–PPA_HT_ (cm^−1^)	Cl–PPA_HH_ (cm^−1^)	PPA1_HT_ (cm^−1^)	PPA16_HT-HH_ (cm^−1^)
*γ*(C–H)	795	774	763	759	828	828
*ν*(O–CH_3_)	1039	1039	1024	1032	1032	1032
*ν*(C–N–C)	1216	1228	1223	1235	1109	1109
*ν*(C–O)	1230	1230	1233	1244	1173	1173
*ν*(C–O–CH_3_)	1242	1249	1253	1257	1250	1250
*ν*(C=C)	1337	1339	1336	1324	1358	1344
*ν*(CH_3_)	1454	1454	1447	1454	1418	1418
Quinoid (Q)	–	–	1537	1513	1490	1490
Benzenoid (B)	1606	1606	1596	1602	1516	1516
*ν*(C–H)*_ring_*	3080–3138	3085–3159	3090–3156	3101–3153	2836	2836
*ν*(N–H)	3515–3518	3427–3514	2101 and 2300	2491–2841	2905–3006	2905–3006

**Table 5:** Tuning parameters using the equivalent circuit model (R_1_, R_2_, C_1_, C_PE_). *R_t_* and *ρ* parameters were calculated using the adjustment results.

Sample	R_1_ (MΩ)	R_2_ (MΩ)	R_t_ (MΩ)	C_1_ (µF)	C_PE1-T_	C_PE1-T_	ρ (MΩcm)	σ (S·cm^−1^)
PPA1_HT_	3.34	77.31	80.65	6.96 × 10^−5^	3.60 × 10^−5^	0.90	8.23 × 10^2^	1.00 × 10^−9^
PPA16_HT-HH_	30.9	2.89 × 10^4^	289.9 × 10^4^	5.65 × 10^−5^	6.09 × 10^−5^	0.98	2.56 × 10^7^	3.90 × 10^−14^

## Data Availability

The data used to support the findings of this study are available from the corresponding author upon request.

## Sample Availability

Samples of the compounds are not available from the authors.
